# Psychological well‐being as a motive for and result of post‐bariatric body contouring procedures

**DOI:** 10.1002/osp4.719

**Published:** 2023-11-01

**Authors:** Britta Pehlke, Filipa Oliveira, Charalampos Varnava, Fabian Nehls, Philipp Wiebringhaus, Maximilian Kueckelhaus, Tobias Hirsch, Alexander Frederik Dermietzel

**Affiliations:** ^1^ Division for Plastic Surgery Department of Trauma, Hand and Reconstructive Surgery University Hospital Muenster Muenster Germany; ^2^ Plastic, Reconstructive, and Aesthetic Surgery, Hand Surgery Fachklinik Hornheide Muenster Germany; ^3^ Department for Plastic and Reconstructive Surgery Institute for Musculoskeletal Medicine Westfaelische Wilhelms‐University Muenster Muenster Germany

**Keywords:** body contouring procedures, body image, depression, psychological well‐being, self‐esteem

## Abstract

**Objective:**

This study investigates whether psychological well‐being in post‐bariatric patients seeking body contouring procedures differs from those who do not seek body contouring procedures, those who have already undergone body contouring procedures, and those who are unsure about body contouring procedures.

**Methods:**

An anonymous, nonrandomized, cross‐sectional survey study was designed. Psychological well‐being of four groups of post‐bariatric‐patients (undergone body contouring procedures, seeking body contouring procedures, not seeking body contouring procedures, unsure about body contouring procedures) were compared.

**Results:**

A total of 345 patients were included in this study. No significant difference between patients seeking body contouring procedures and those not seeking body contouring procedures was found with regard to depressive symptoms, self‐esteem, and body image. Patients who had already undergone body contouring procedures scored lower on depressive symptoms (*p* = 0.035) and reported feeling more attractive (*p* < 0.001) and less insecure (*p* = 0.030) than patients who had not yet undergone body contouring procedures but sought it. Satisfaction with the result of the body contouring procedures was associated with lower depression levels (*p* < 0.001), higher self‐esteem (*p* < 0.001) and a more positive body‐image (*p* < 0.001).

**Conclusions:**

Depressive symptoms or low self‐esteem are not motivational factors for post‐bariatric patients to seek body contouring procedures. Body contouring procedures are associated with improvement in psychological well‐being in post‐bariatric patients. Patients' satisfaction with the result of the body contouring procedures is significantly associated with positive psychological well‐being.

## Graphical abstract

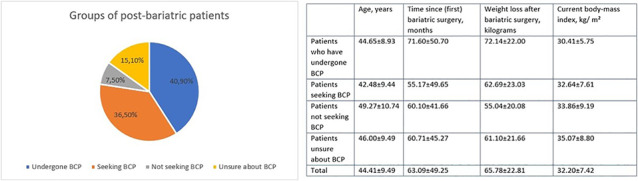

## 1 INTRODUCTION

About one out of eight adults worldwide is directly affected by obesity,^1^ with numbers expected to continue to increase dramatically further in the upcoming years. Only a small proportion of people with obesity achieve a substantial weight loss by means of lifestyle modification and pharmacotherapy.^2^ One male patient out of 210 with grade I obesity succeeds in achieving and maintaining normal weight, and the quota for females is one to 124.^3^ The chance of achieving normal weight further decreases as the body mass index (BMI) increases. Bariatric surgery is currently the most effective procedure for achieving considerable and long‐lasting weight loss in patients with obesity.^4^ These surgical interventions result in a significant number of patients experiencing massive weight loss. Weight loss has a positive impact on physical comorbidities such as diabetes, hypertension, and orthopedic complaints.^5^

Obesity is strongly associated with a number of psychological comorbidities.^6^ People with obesity have a 55% increased risk of developing depression.^7^ Positive associations between obesity and anxiety disorders,^8^ binge‐eating‐disorder,^9^ attention‐deficit/hyperactivity disorder (ADHD),^10^ and borderline personality disorder^11^ have also been found in previous studies. This relationship becomes stronger as the patients' body‐mass‐index increases.^12^ The causality is largely unknown, but the relationship is most likely bidirectional.^13^ Previous literature has been inconclusive with regard to the influence preoperative psychological functioning has on post‐operative psychological health and subsequent weight loss.^14^ However, patients undergo numerous changes in psychological well‐being after bariatric surgery.

Numerous improvements in psychological well‐being and functioning are observed in patients after bariatric surgery.^15^ Shortly after bariatric surgery and subsequent weight loss, depression and anxiety significantly decrease^16^, ^17^ whereas body image significantly improves.^18^ Previous studies also found an improvement in social relations and employment status^19^ as well as an improved quality of life^20^ in post‐bariatric patients. Bariatric surgery results in a significant improvement in the quality of sexual life,^21^ sexual functioning and relationship quality^22^ in women. However, improvement in psychological health and well‐being declines about 2 years after patients have undergone bariatric surgery.^23^ This effect may be caused by patients' perceived impaired quality of life and dissatisfaction with their physical appearance^24^ as they frequently report suffering from excess skin after massive weight loss.^25^

Massive weight loss patients experience after bariatric surgery often results in excess skin, causing functional and health impairments such as inflammation and fungal infection. The most commonly affected body areas are the abdomen, upper arms, and inner thighs.^26^ This can lead to a decreased feeling of well‐being and self‐esteem in post‐bariatric patients.^27^ By performing body contouring procedures (body contouring procedures), the surplus of skin can be removed and body proportions restored. Previous studies and reviews showed significant improvement in post‐bariatric patients' quality of life after body contouring procedures.^28^, ^29^

Previous studies investigated differences in the psychological well‐being of post‐bariatric patients before and after body contouring procedures.^30^, ^31^ Results showed a significant decrease in symptoms of depression after body contouring procedures.^32^ Patients also reported an improved quality of life^33^ and an enhanced psychological well‐being^34^ and social functioning.^35^ Furthermore, body contouring procedures were found to be associated with the improvement of weight loss^36^ and patients who underwent body contouring procedures reported higher satisfaction with their appearance.^37^

Prior research found the desire for body contouring procedures to be as high as 73% for adult post‐bariatric patients.^38^ However, the rate of patients eventually undergoing body contouring procedures is significantly lower, with numbers ranging from 7% to 25% depending on the country of origin and demographics.^39^, ^40^, ^41^ These discrepancies were found to be rarely due to rejection by surgeons (because of unstable weight, a high BMI, the lack of functional impairments or smoking habits) or because of patients' withdrawal. In most cases, the lack of cost coverage was the reason for not undergoing body contouring procedures eventually.^42^

The aim of the current study was to compare self‐esteem, body image and level of depression among post‐bariatric patients according to their willingness to undergo body contouring procedures, and according to patients' satisfaction with the results of body contouring procedures.

## 2 METHODS

In a nonrandomized, cross‐sectional study, a link to an anonymous online survey was distributed to post‐bariatric patients through obesity organizations, obesity self‐help groups, hospitals, obesity centers, and obesity‐ and bariatric surgery‐related groups on social media platforms between April and May 2022. Inclusion criteria were age over 18 years and a minimum of 2 years after their (first) bariatric surgery. Informed consent was given by all participants. Data collection was performed using SoSciSurvey. Statistical analyses were conducted using the IBM Statistical Package for Social Sciences version 28.

### 2.1 Instruments

Depressive symptoms The level of depressive symptoms was measured using the German version of the simplified Beck Depression Inventory (BDI‐V).^43^ This represents an economic and user‐friendly version of the well‐established and validated BDI‐V.

### 2.2 Self‐esteem

Self‐esteem was measured using the Rosenberg Self‐esteem scale.^44^ The Rosenberg Self‐esteem scale is an instrument established to measure global self‐esteem defined as the entirety of an individual's positive and negative attitudes toward themselves. The scale comprises 10 statements to comprehend the entirety of positive and negative aspects of an individual's self‐esteem. Participants indicated in how far these statements applied to them on a 6‐point scale ranging from 1 = totally disagree to 6 = totally agree. The scale postulates self‐esteem as a unidimensional construct.

### 2.3 Body image

Body image was assessed using the Questionnaire for the evaluation of the body (FBeK).^45^ This instrument consists of 52 items which can be grouped into three subscales “Insecurity/parathesia” (19 items), “Attractiveness/self‐confidence” (13 items) and “Accentuation of the body/sensibility” (20 items). Participants indicated dichotomously whether they agreed with the statements or not.

### 2.4 Satisfaction with the result of body contouring procedures

The satisfaction with the result of the surgery was measured by a single item (“How satisfied are you with the result of the body contouring procedure?”). Answer options ranged on a 6‐point scale from 1 = “totally unsatisfied” to 6 = “totally satisfied”. Branching logic was used to only present this question to patients who had already undergone body contouring procedures. If they had undergone more than one body contouring procedure, the question applied to the most recent surgery undergone.

### 2.5 Sociodemographic and obesity‐related data

In addition to sociodemographic data (sex, age), data on weight and height of the patients were collected for the computation of the body‐mass index. Furthermore, patients were asked to report the date of their (first) bariatric surgery and the subsequent weight loss.

Patients were divided into four groups according to whether they had already undergone body contouring procedures, planned to undergo body contouring procedures, did not seek body contouring procedures, or were unsure in this regard.

### 2.6 Ethics and statistics

Due to the completely anonymous and non‐randomized design of the survey study, according to the IRB, no ethical approval was required. The design of the study did not allow deduction of any personal information or identity from the participants. Furthermore, participants were only able to access the survey after being provided with written information on the aim and content of the study and after informed consent was obtained. The results of the trial are presented as mean and standard deviation when appropriate. Comparisons between the four patient groups were made using a one‐way ANOVA and posthoc Tamhane tests. Statistical significance was set at *p* < 0.05 (2‐sided). Correlations between variables were analyzed using Spearman's rank correlation test.

## 3 RESULTS

### 3.1 Patient characteristics

Six hundred one participants accessed the survey. 345 participants (90.4% female, 9.6% male) filled in the survey completely, fulfilled the inclusion criteria and were included in the study. The mean age of the participants was 44.41 ± 9.49 years, ranging from 25 to 77 years. The average time since the (first) bariatric surgery was 63.09 ± 49.25 months. On average, patients lost 65.78 ± 22.81 kg after bariatric surgery. At the time of participating in the study, the average body‐mass index was 32.20 ± 7.43 (Table 1).

**TABLE 1:** Patient characteristics by subgroup of post‐bariatric patients.

	Age, years	Time since (first) bariatric surgery, months	Weight loss after bariatric surgery, kilograms	Current body‐mass index, kg/m^2^
Patients who have undergone BCP	44.65 ± 8.93	71.60 ± 50.70	72.14 ± 22.00	30.41 ± 5.75
Patients seeking BCP	42.48 ± 9.44	55.17 ± 49.65	62.69 ± 23.03	32.64 ± 7.61
Patients not seeking BCP	49.27 ± 10.74	60.10 ± 41.66	55.04 ± 20.08	33.86 ± 9.19
Patients unsure about BCP	46.00 ± 9.49	60.71 ± 45.27	61.10 ± 21.66	35.07 ± 8.80
Total	44.41 ± 9.49	63.09 ± 49.25	65.78 ± 22.81	32.20 ± 7.42

**FIGURE 1:**
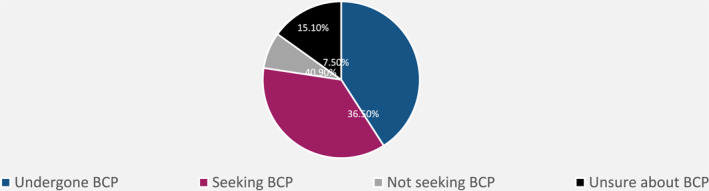
Groups of post‐bariatric patients.

Forty point nine percent (*N* = 141) of the patients had already undergone body contouring procedures, 36.5% (*N* = 126) intended to undergo body contouring procedures, 15.1% (*N* = 52) were unsure about undergoing body contouring procedures and 7.5% (*N* = 26) of the patients excluded the option of body contouring procedures for themselves. The four subgroups were regarded as independent (Figure 1).

A one‐way ANOVA revealed that there was no statistically significant difference in average time since (first) bariatric surgery between the groups (*F* (3, 341) = 2.595, *p* = 0.052).

A one‐way ANOVA revealed that there was a statistically significant difference in average weight loss between at least two groups (*F* (3, 330) = 7.369, *p* < 0.001). A posthoc Tamhane test showed that patients who had already undergone body contouring procedures lost more weight than patients who intended to undergo body contouring procedures (*p* = 0.006), patients who did not intend to undergo body contouring procedures (*p* = 0.002) and those who were unsure whether they wanted to undergo body contouring procedures (*p* = 0.015). No significant difference was found between the other three groups.

A one‐way ANOVA revealed that there was a statistically significant difference in the current BMI between at least two groups (*F* (3, 332) = 6.080, *p* < 0.001). A posthoc Tamhane test showed that patients who had already undergone body contouring procedures had a significantly lower BMI than those who were unsure whether they wanted to undergo body contouring procedures (*p* = 0.004). No significant difference was found between the other three groups.

A one‐way ANOVA revealed that there was a statistically significant difference in the average age between at least two groups (*F* (3, 341) = 4.661, *p* = 0.003). A posthoc Tamhane test showed that patients who intended to undergo body contouring procedures were on average younger than patients who did not intend to undergo body contouring procedures (*p* = 0.031). No significant difference was found between the other three groups.

### 3.2 Depressive symptoms

**FIGURE 2:**
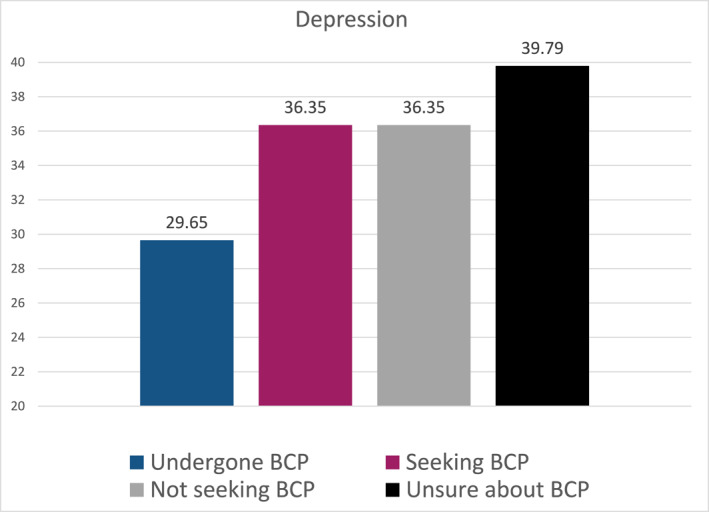
Mean differences in depression scores.

A one‐way ANOVA revealed that there was a statistically significant difference in mean depressive symptoms score between at least two groups (*F* (3, 341) = 4.169, *p* = 0.006) (Figure 2).

A posthoc Tamhane test showed that patients who had already undergone body contouring procedures (*M* = 29.65, SD = 18.77) reported less depressive symptoms than patients who intended to undergo body contouring procedures (*M* = 36.35, SD = 20.41) (*p* = 0.035) and those who were unsure whether they wanted to undergo body contouring procedures (39.79, SD = 22.74) (*p* = 0.031). No significant difference was found between patients who intended to undergo body contouring procedures and those who did not (*M* = 36.35, SD = 24.61).

### 3.3 Self‐esteem

**FIGURE 3:**
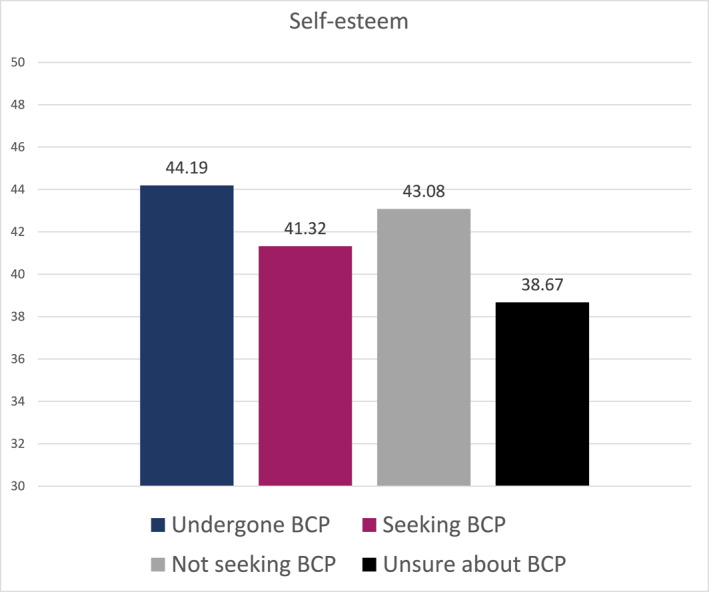
Mean differences in self‐esteem.

A one‐way ANOVA revealed that there was a statistically significant difference in the mean self‐esteem score between at least two groups (*F* (3, 341) = 3.509, *p* = 0.016) (Figure 3). A posthoc Tamhane test showed that patients who had undergone body contouring procedures reported higher self‐esteem (*M* = 44.19, SD = 10.23) than patients who were unsure about their intention to undergo body contouring procedures (*M* = 38.67, SD = 12.40) (*p* = 0.031). With regard to reported self‐esteem, no significant difference was found between post‐bariatric patients who intended to undergo body contouring procedures (*M* = 41.32, SD = 11.08) and those who did not (*M* = 43.08, SD = 14.19). Furthermore, patients who intended to undergo body contouring procedures did not differ from those who had undergone body contouring procedures in terms of self‐esteem.

### 3.4 Body image

The body image measured by the “Questionnaire for the evaluation of the body” can be divided into three subscales.

**FIGURE 4:**
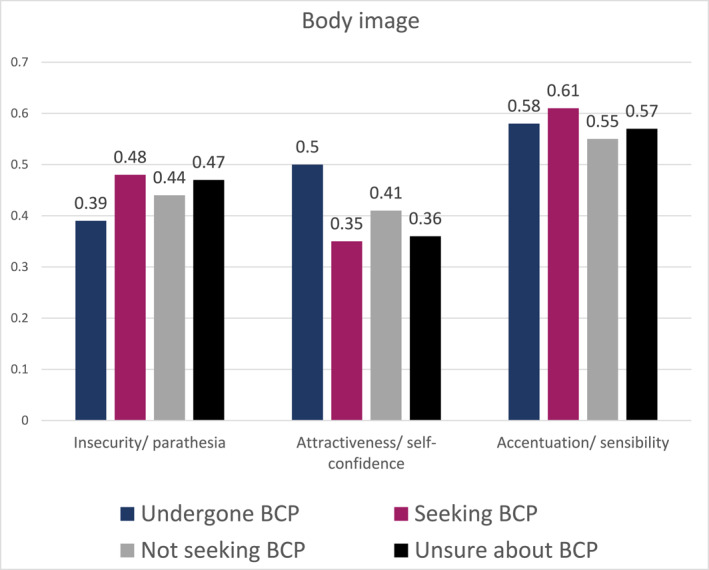
Mean differences in body image (divided into three subscales).

A one‐way ANOVA revealed that there was a statistically significant difference in the mean of the subscale of body image “Insecurity/parathesia” between at least two groups (*F* (3, 338) = 2.906, *p* = 0.032) (Figure 4). A posthoc Tamhane test revealed that patients who had undergone body contouring procedures (*M* = 0.39, SD = 0.24) felt less insecure about their outer appearance than patients who sought body contouring procedures (*M* = 0.48, SD = 0.25) on the subscale “Insecurity/parathesia” (*p* = 0.003). No significant difference was found between post‐bariatric patients who sought body contouring procedures and those who did not want to undergo body contouring procedures (*M* = 0.44, SD = 0.27).

A similar pattern was found with regard to the subscale “Attractiveness/self‐confidence”. A one‐way ANOVA revealed that there was a statistically significant difference in the mean of the subscale of body image “Attractiveness/self‐confidence” between at least two groups (*F* (3, 337) = 5.660, *p* < 0.001). A posthoc Tamhane test showed that patients who had already undergone body contouring procedures felt more attractive and reported higher self‐confidence (*M* = 0.50, SD = 0.29) than patients who planned to undergo body contouring procedures (*M* = 0.35, SD = 0.28) (*p* < 0.001). Post‐bariatric patients who wanted to undergo body contouring procedures and those who did not want to undergo body contouring procedures (*M* = 0.41, SD = 0.36) did not differ significantly on this dimension.

A one‐way ANOVA revealed that there was no statistically significant difference in mean of the subscale of body image “Accentuation/sensibility” between the four groups (*F* (3, 341) = 1.441, *p* = 0.231).

### 3.5 Time since bariatric surgery and psychological well‐being

There was a significant correlation between the time since bariatric surgery and depression (*r* = 0.20, *p* < 0.001), self‐esteem (*r* = −0.12, *p* = 0.033). The time since bariatric surgery was also associated with two subscales of body image, insecurity/parathesia (*r* = 0.20, *p* < 0.001) and attractiveness/self‐confidence (*r* = −0.17, *p* < 0.001). The longer the time since the bariatric surgery, the poorer the psychological well‐being was.

No correlation was found between weight loss and any of the aspects of psychological well‐being.

### 3.6 Satisfaction with the result of body contouring procedures

Patients who had undergone body contouring procedures (*N* = 141) reported high satisfaction with the obtained result (*M* = 4.8 ± 1.1).

The patient's satisfaction with the results of the body contouring procedures displayed a significant (negative) correlation with depression (*r* = −0.55, *p* < 0.001), self‐esteem (*r* = −0.55, *p* < 0.001) and each of the three subscales of body image. Insecurity/parathesia (*r* = −0.47, *p* < 0.001) and accentuation/sensibility (*r* = −0.22, *p* = 0.009) were negatively associated, attractiveness/self‐confidence (*r* = 0.44, *p* < 0.001) was positively associated with patients' satisfaction with the results of the body contouring procedures. Post‐bariatric patients felt less depressed and experienced higher self‐esteem and a more positive body image the more satisfied they were with the result of the body contouring procedures.

## 4 DISCUSSION

The results of the current study shed a critical and differentiated light on patients' psychological well‐being in the context of post‐bariatric body contouring procedures. The assumption that patients seeking body contouring procedures suffer from more psychological strains such as depressive symptoms, lower self‐esteem and poorer body image than patients who do not seek body contouring procedures was refuted. The two groups did not differ in any of the investigated aspects of psychological well‐being and health. In contrast, significant differences with regard to depressive symptoms, self‐esteem, and body image were found between post‐bariatric patients before and after body contouring procedures. These results allow the conclusion that^1^ post‐bariatric patients seeking body contouring procedures do not display poorer psychological health than patients who do not want to undergo body contouring procedures and^2^ body contouring procedures play are associated with psychological well‐being in post‐bariatric patients.

The “German Bariatric Surgery Registry” (GBSR) contains data from about 92% of all bariatric procedures conducted in Germany from 2005 on. The registry reports an average age of 42.70 years with women being much more likely to undergo bariatric surgery (71.50%) than men (28.50%), which is not identical but similar to the distributions in the present study. The average weight loss reported in the German bariatric Surgery Registry is between 61.90 and 69.50 kg, depending on the bariatric procedure performed^46^, comparable to the average weight loss of 65.78 kg in the present study. It can thus be assumed that the patients in the current study represented the population of German post‐bariatric patients well.

The fact that patients who had already undergone body contouring procedures displayed a significantly greater weight loss than the other three groups raises the question of how far weight loss itself may contribute to improved psychological well‐being and vice versa.

The results of the current study do not allow causal conclusions. A randomized experimental design seems difficult to establish in the context of body contouring procedures. It seems feasible to conduct longitudinal studies to evaluate the interaction of patient‐ and environmental related factors as well as medical and psychological factors from prior to bariatric surgery up to the time following potential body contouring procedures. In this context, subgroups should be studied, such as patients with lipedema. Lipedema is known to be associated with obesity^47^ and psychological health.^48^ Examining the influence of lipedema on psychological health of post‐bariatric patients can provide further insights into how interdisciplinary treatment can help patients all encompassing.

The anonymous and online‐based design of the present study enabled the inclusion of post‐bariatric patients who do not want to undergo body contouring procedures and a subsequent comparison of this group with other groups of post‐bariatric patients. This design has drawbacks, though. It makes it difficult to control for factors, such as whether and to what extent patients are under supervision of an obesity center, which may also influence relevant outcomes. A comparison of patients from obesity centers of different sizes and levels of experience can lead to further insights in future studies.

The data collected in the study were based on subjective self‐reports by the patients. This is relevant as psychological well‐being is based solely on subjective perceptions. The fact that the study was conducted as an online survey potentially reduced the participants' tendency for socially desired answers. In the case of post‐bariatric patients, a sensitive approach to this matter is especially important as they might be hesitant to communicate openly with physicians and surgeons for fear of saying something that might reduce their chance of having costs for body contouring procedures covered. An anonymous setting made patients probably more willing to openly indicate their state of psychological well‐being. In future studies, physicians' impression can be integrated to reveal possible discrepancies in perceptions and to optimize communication between them and their patients.

A sole focus on physical impairments and diseases does not comply with the complex and diverse health condition of post‐bariatric patients. The basic recommendation to rather seek psychotherapy than undergoing body contouring procedures is not expedient. Rather, both interventions should be regarded as supplementary options which can contribute substantially to the patients' physical and psychological health. A prerequisite is the interdisciplinary collaboration between all different experts involved in the treatment of the patients prior to and after bariatric surgery. This is in accordance with the latest guidelines issued in 2022 by the two leading authorities of bariatric and metabolic surgery, the American Society of Metabolic and Bariatric Surgery and International Federation for the Surgery of Obesity and Metabolic Disorders (IFSO).^49^ They recommend a holistic approach integrating all involved medical and psychological disciplines as well as an individualized consultation of the post‐bariatric patients with regard to the most appropriate therapies for them.

The fact that only 7.5% of patients do not consider undergoing body contouring procedures and 15.10% report to be unsure whether they want to undergo body contouring procedures shows the need for consultation by a plastic surgeon as part of the preparation for bariatric surgery and post‐bariatric aftercare. The current study is the first to not only include post‐bariatric patients who seek body contouring procedures or have already undergone it but also those patients who do not want to undergo body contouring procedures or are unsure about it. However, this is necessary to fully understand post‐bariatric patients' motivational factors and needs with regard to body contouring procedures. A consultation as early as prior to bariatric surgery can inform patients about the excess skin and possible subsequent impairments. It can also prevent unrealistic expectations toward plastic surgery after massive weight loss and enable patients to evaluate their personal costs and benefits associated with body contouring procedures. This is crucial with regard to the significant association found in the current study between the satisfaction with the body contouring procedures and each of the aspects of psychological well‐being. This evidence indicates the potential of body contouring procedures as part of an interdisciplinary and holistic treatment concept of obesity to contribute to patients' overall health. Furthermore, early consultation by a plastic surgeon helps patients with accomplishing all requirements for applying for cost coverage of body contouring procedures on time.

The results indicated no significant relationship between weight loss and psychological well‐being. However, the time since bariatric surgery is negatively related to all but one assessed aspects of psychological well‐being. This supports the assumption that the excess skin and associated impairments might have a negative influence on psychological health in the long‐term and should be addressed in future longitudinal studies.

Previous research^50^ and reviews^51^, ^52^ pointed out the importance of considering psychological aspects in the context of body contouring procedures. The present research builds on these findings by incorporating plastic surgery and psychology—two disciplines which are still underrepresented in obesity research and treatment. A successful and lasting treatment of obesity prior to and after bariatric surgery not only requires a decrease in physical comorbidities but also an improvement in mental health and psychological well‐being. The reduction or even absence of complaints and problems in each of both aspects is a precondition for the patients to re‐gain their health and be able to engage fully in family, social, and occupational life.

The results showed significant differences between post‐bariatric patients before and after body contouring procedures. The assumption that patients seeking body contouring procedures experience more psychological strains such as depression, lower self‐esteem, and poorer body image than patients who do not seek body contouring procedures was not supported by the data obtained in this study. Although the current study does not allow to draw any causal conclusions, body contouring procedures seem to be associated with patients' psychological well‐being. Future studies should investigate which groups of post‐bariatric patients benefit most from body contouring procedures.

## AUTHOR CONTRIBUTIONS

Author contributions Tobias Hirsch, Alexander Dermietzel and Britta Pehlke initiated and conceptualized the study. Britta Pehlke and Alexander Dermietzel performed the statistical analyses and interpretation of the data, and Britta Pehlke wrote the first draft of the manuscript. Tobias Hirsch contributed to the design of the study. Filipa Oliveira, Charalampos Varnava, Fabian Nehls, Philipp Wiebringhaus and Maximilian Kueckelhaus contributed to the interpretation of the data, contributed to the further writing of the manuscript and approved the final version.

## CONFLICT OF INTEREST STATEMENT

The authors declare no conflicts of interest.
